# Decoding brain aging trajectory: predictive discrepancies, genetic susceptibilities, and emerging therapeutic strategies

**DOI:** 10.3389/fnagi.2025.1562453

**Published:** 2025-03-19

**Authors:** Yulia Komleva, Kristina Shpiliukova, Nikolai Bondar, Alla Salmina, Elena Khilazheva, Sergey Illarioshkin, Michael Piradov

**Affiliations:** ^1^Research Center of Neurology, Moscow, Russia; ^2^Laboratory of Molecular Virology, First Moscow State Medical University (Sechenov University), Moscow, Russia; ^3^Department of Biological Chemistry with Courses in Medical, Research Institute of Molecular Medicine and Pathobiochemistry, Pharmaceutical and Toxicological Chemistry Prof. V.F. Voino-Yasenetsky Krasnoyarsk State Medical University of the Ministry of Healthcare of the Russian Federation, Krasnoyarsk, Russia

**Keywords:** brain aging, neuroinflammation, individual aging trajectory, cell senescence, cell competition, delayed and accelerated aging, gene, digital twin

## Abstract

The global extension of human lifespan has intensified the focus on aging, yet its underlying mechanisms remain inadequately understood. The article highlights aspects of genetic susceptibility to impaired brain bioenergetics, trends in age-related gene expression related to neuroinflammation and brain senescence, and the impact of stem cell exhaustion and quiescence on accelerated brain aging. We also review the accumulation of senescent cells, mitochondrial dysfunction, and metabolic disturbances as central pathological processes in aging, emphasizing how these factors contribute to inflammation and disrupt cellular competition defining the aging trajectory. Furthermore, we discuss emerging therapeutic strategies and the future potential of integrating advanced technologies to refine aging assessments. The combination of several methods including genetic analysis, neuroimaging techniques, cognitive tests and digital twins, offer a novel approach by simulating and monitoring individual health and aging trajectories, thereby providing more accurate and personalized insights. Conclusively, the accurate estimation of brain aging trajectories is crucial for understanding and managing aging processes, potentially transforming preventive and therapeutic strategies to improve health outcomes in aging populations.

## Introduction

It is well known, that human life span has increased around the world the last few 100 years. In countries with low mortality, the average has risen to around 87 years (Aburto et al., 2020). According to the data, the aging population will reach 2,1 billion people by 2050 (Wyss-Coray, 2016). Due to large quantity of elderly people means that there is a need to improve their quality of life. Moreover, there is a risk of age-related problems such as neurodegenerative diseases (e.g., Parkinson’s (PD) and Alzheimer’s (AD), etc.). These disorders caused by progressive neuronal death, which underlies their clinical manifestation and lead to cognitive decline and to the overall decline in neurological function. According to the latest research, one in ten people has this type of brain damage (in people over 65 years) (Hou et al., 2019; Azam et al., 2021). The high prevalence of neurodegeneration is the main reason for scientific research into its causes and progression (Azam et al., 2021).

Despite aging being a nearly universal phenomenon, its underlying causes remain poorly understood. Various aging phenotypes have been observed, and numerous hypotheses have been proposed (Kirkwood, 1977; Gladyshev, 2013; López-Otín et al., 2013). A series of studies have identified nine fundamental mechanisms that contribute to the aging process, including telomere shortening, genetic mutations, mitochondrial dysfunction, cellular damage, epigenetic changes, disruption of protein homeostasis, chronic inflammation, metabolic disorder and stem cell exhaustion (López-Otín et al., 2013; Zia et al., 2021; Guo et al., 2022). Each of these interactions is distinguished by its own unique markers (Blinkouskaya et al., 2021).

The connection between these factors is complex, leading to the determination of how individuals age and their susceptibility to age-related diseases. Adopting a healthy lifestyle can be helpful in promoting healthy aging and improving overall well-being, even although aspects of aging are beyond our control (Wang et al., 2022). There are several conventional methods for estimating biological age (BA) (Box 1). Currently, no widely accepted method exists for detecting BA using biomarkers. Mostly used methods for estimating BA are the multiple linear regression (MLR), the Hochschild’s method, the principal component analysis (PCA) and the Klemera and Doubal’s method (KDM) (Jia et al., 2017). However, the estimation of BA through physiological biomarkers is becoming increasingly prevalent (Husted et al., 2020). Nevertheless, the specific cellular and molecular mechanisms driving aging and their variations across different biological contexts are still unknown (Azam et al., 2021; Zia et al., 2021; Guo et al., 2022). Understanding the fundamental nature of aging and its detrimental effects drives the need to identify why some people age faster and neurons become non-resistant to stress, and some people remain functionally intact for a very long time, exhibiting delayed aging (Kimmel et al., 2019).

**Figure 1:**
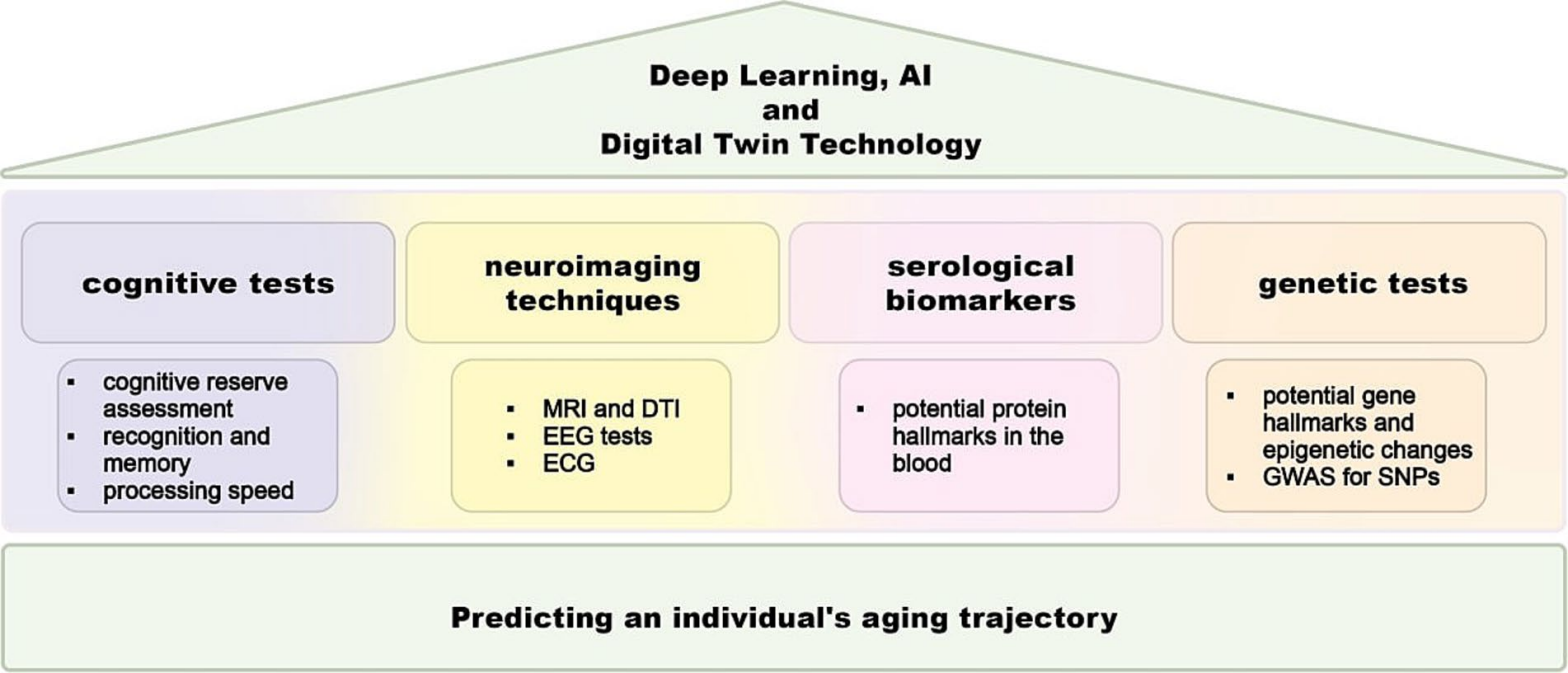
Scheme for brain aging gap assessment. A comprehensive approach for assessing the brain aging gap, which involves the integration of free four primary methods: cognitive tests, neuroimaging systems, and biomarker analysis. Neuroimaging techniques domain employs advanced imaging techniques to evaluate brain structure and function. Genetic analysis involves the examination of genetic variations and their association with brain aging. Serological assay identifies the pull of proteins that are related to brain aging gap. The cognitive test domain involves a series of standardized tests designed to assess various cognitive functions, such as memory, attention, and executive function. Deep learning and artificial intelligence algorithms help in predicting brain aging scores by synthesizing complex datasets and enhancing the accuracy and reliability of brain aging assessments. MRI, magnetic resonance imaging; DTI, diffusion tensor imaging; EEG, electroencephalography, ECG, electrocardiography; GWAS, genome-wide association studies; SNP, single-nucleotide polymorphism; AI, artificial intelligence.

Estimating brain age using a predictive model trained on neuroimaging data from a large cohort of healthy individuals is a valuable method for identifying personalized markers of potential future cognitive impairments (Zoubi et al., 2018; Jonsson et al., 2019). By establishing the normal aging trajectory in healthy older adults, this method helps in identifying clinically significant deviations. Research using this technique has shown accelerated brain aging in patients with AD, traumatic brain injury, human immunodeficiency virus, and schizophrenia. The predicted age difference (PAD), which is the difference between predicted brain age and chronological age, indicates the degree of deviation from the healthy aging trajectory and has been linked to cognitive impairment and the risk of dementia (Figure 1) (Jonsson et al., 2019). Previous studies have also found that brain-aging trajectories are associated with cognitive impairments, with factors such as apolipoprotein E (APOE) ε4 and amyloid *β* influencing these trajectories in the context of AD (Huang et al., 2021). Nevertheless, a contemporary examination of the latest biomarkers and genes is necessary to identify deviations from the standard trajectory of brain aging (Hou et al., 2019; Mantovani et al., 2020).

Over the past 50 years, substantial individual variations have been observed in the progression of cognitive aging as well as in the age-related alterations in brain structure and function. Several theoretical constructs have been proposed to account for this phenomenon of resilience, including brain reserve, cognitive reserve, brain maintenance, and compensation (Stern et al., 2019). Extensive research has documented aging phenotypes at the physiological level, highlighting functional and behavioral markers of age-related decline. Similarly, molecular profiling of nucleic acids, proteins, and metabolites has provided detailed phenotypic descriptions of aging within specific tissues. Different “omics” approaches have uncovered common changes, such as increased inflammatory pathway activity across various tissues (Benayoun et al., 2019), alterations in immunological and stress response pathways in multiple organs (Angelidis et al., 2019) as well as changes in fatty acid profiles across several tissues (Houtkooper et al., 2011).

The present review is motivated by the advances in studying of opposite trajectories, namely normal aging and frailty as an accelerated aging phenotype and following age-related brain-specific genetic- and cell-level changes. To aid in understanding these concepts, we provide a short glossary defining key terms related to different types of age and accelerated aging (Box 1).

BOX 1 **Main terms in aging trajectory.**

*Chronological age* — the amount of time that has elapsed from birth to a given date and is the main way of defining age (Husted et al., 2020).

*Biological age* (also known as physiological or functional age) — an assessment of age based on the physiological state of an individual’s body. Biological aging occurs as a person gradually accumulates damages to various cells, it considers not only the time elapsed, but also a number of different biological and physiological developmental factors, such as genetics, lifestyle, nutrition and comorbidities (Husted et al., 2020).

*Predictive brain aging* — the estimated age of the nervous system, determined by evaluating neurological and cognitive changes (Zoubi et al., 2018). This assessment uses brain imaging data, cognitive ability tests, and specific genetic biomarkers to gauge how the brain is aging.

*Predicted age difference* — the discrepancy between predictive brain aging (or biological age) and chronological age (Jonsson et al., 2019; Huang et al., 2021). A larger predicted age difference may indicate a higher risk of age-related diseases, including neurodegenerative conditions.

*Accelerated aging* — the rapid progression of the aging process, leading to the early onset of multiple age-related changes and health issues (Gonneaud et al., 2021). It signifies that an individual’s biological age is advancing faster than their chronological age.

*Frailty* — a clinical state characterized by the increased vulnerability of an individual to stressors caused by age-related cumulative decline in multiple physiological systems, an accelerated aging phenotype (Sugimoto et al., 2022).

*Cognitive frailty* — the simultaneous presence of physical frailty and cognitive impairment in older individuals without a definite diagnosis of dementia; an intermediate stage between normal aging and accelerated aging. This condition is potentially reversible and highlights the need for early interventions to prevent further decline (Mantovani et al., 2020).

*Individual aging trajectory* — a personalized approach to managing and explain variations in aging in individuals to promote healthy aging. It involves tailoring strategies to enhance lifespan and quality of life based on an individual’s unique characteristics and genetic predispositions (Huang et al., 2021; Shen et al., 2022).

We review the study of brain gene expression related to two distinct phenotypes of brain health: accelerated aging and delayed aging. Accelerated aging involves genes associated with a rapid decline in brain health (Gonneaud et al., 2021), while delayed aging includes candidate genes linked to essential brain functions and longevity (Table 1). This review aims to elucidate the key molecular and cellular mechanisms defining individual brain aging trajectories, focusing on the interplay between genetic susceptibilities, impaired bioenergetics, neuroinflammation, and cellular senescence. We analyze how disruptions in mitochondrial function, metabolic pathways, and stem cell exhaustion contribute to accelerated brain aging, emphasizing their role in neurodegenerative diseases. Additionally, we explore methods for estimating biological and brain age, including neuroimaging, cognitive assessments, and genetic profiling, with a particular focus on predictive models that distinguish between normal and pathological aging. By integrating emerging technologies such as artificial intelligence and digital twins, we highlight the potential for refining aging assessments and developing targeted therapeutic strategies. Ultimately, this review seeks to bridge the gap between fundamental aging research and clinical applications, offering insights into personalized interventions that could mitigate cognitive decline and enhance brain resilience during aging.

**Table 1:** Non-pathogenic functions of key genes and proteins.

Gene	Functions	References
*APOE4*	Normal catabolism of triglyceride-rich lipoproteins; control and regulation of lipoprotein metabolism	Huebbe and Rimbach (2017)
*DNM1*	GTPase which is involved in endocytosis and recycling of synaptic vesicles; mediates the uptake of synaptic vesicles	Dhindsa et al. (2015)
*SYN1*	Control of synaptic endocytosis; synaptic plasticity and transmission	Baldelli et al. (2007)
*SYN2*	Neurotransmission	Medrihan et al. (2013)
*mTOR*	Regulation of mRNA translation; regulation of protein synthesis; regulation of cellular response on hypoxia; early brain development	Lipton and Sahin (2014)
*RAGE*	Recruiting immune cells	Chavakis et al. (2004)
*NF-κB*	DNA transcription; cytokine production; cell survival, embryonic development; cellular stress response; neuroprotection, insulin sensitivity; bone resorption	Serasanambati and Chilakapati (2016)
*GSK3β*	Intracellular signaling, gene expression and apoptosis; neurons remodeling and cytoskeletal organization; one of the primary neurons Tau-kinase	Forlenza et al. (2011)
*FOXO*	Regulation of cell cycle and proliferation, apoptosis, autophagy, metabolism and energy homeostasis, oxidative stress resistance, inflammation and cell differentiation; stem cell maintenance, neuroprotection	Wang et al. (2014)
*TNF*	Cell survival, differentiation, proliferation and cell death; activation of NF-κB signaling pathway, immune system regulation; inflammation	Wang and Lin (2008)
*DIMT1*	Cellular stress response; mitochondrial function, insulin secretion and ribosome biogenesis	Shen et al. (2021)
*Drp1*	Mitochondria fission; cell death regulation, metabolism, proliferation and differentiation	Rosdah et al. (2020)
*MFN1/2*	Dynamin-related GTPases; MFN1 - mitochondrial fusion. MFN2 - mitochondria–endoplasmic reticulum contact	Ohta and Nishiyama (2011)
*APP*	Neural maturation and growth during brain development, neurogenesis of neural stem cells; cell fate specification; synapse formation, neural plasticity	Coronel et al. (2018)
*PS1*	Regulation of APP processing; Notch receptor cleavage; neuronal differentiation	Zhu et al. (2017)
*MAPT*	Engagement in microtubule assembly, interaction of microtubules with the cytoskeleton; synaptic plasticity and axonal transport – neuronal structure maintenance	Sündermann et al. (2016)
*KLF3*	Transcriptional factor; negative regulator of adipogenesis	McConnell and Yang (2010)
*KANSL1*	Regulation of gene expression through histone H4 acetylation; chromatin modification	Linda et al. (2020)
*MAPT-AS1*	Natural antisense transcript. Participates in MAPT silencing and preventing pathologies caused by MAPT increased expression.	Simone et al. (2021)
*CRHR1*	Stress response, regulation adrenocorticotropic hormone release; receptor which binds corticotrophin family members	Liu et al. (2016)
*RUNX2*	Osteoblast differentiation and their G1 transition, cell migration, skeletal morphogenesis; some regulation in skeletal gene expression; bone development	Vimalraj et al. (2015)
*NKX6-2*	Central nervous system development, oligodendrocyte and, possible, astrocytes differentiation; regulation of myelination	Cai et al. (2010)
*CDKN1A (p21Cip1)*	Cell cycle regulation for G2/M transition; role in cell differentiation and migration, apoptosis and iPSC reprogramming; DNA damage response	Kreis et al. (2019)
*CDKN2A (p16INK4a)*	Tumor suppression, control of proliferation; engagement in cell cycle G1 progression by phosphorylation of retinoblastoma protein	Witkiewicz et al. (2011)
*MYC/c-Myc*	Regulation of the cell cycle and the control of proliferation during embryogenesis, cell differentiation and apoptosis; inhibiting the negative regulators of cell cycle such as p21; may act as a transcription factor for expression of cell progression’s genes.	Jha et al. (2023)
*Srsf7*	Alternative splicing factor and mRNA transporter; cell viability and growth; regulation of m6A RNA modification that involved in cells differentiation, cancer development, immune system and some cognitive function; RNA splicing	Cun et al. (2023)
*Ezh2*	Cell cycle, cell differentiation, autophagy and apoptosis regulation; DNA damage repair; histone methylation; gene silencing	Liu and Yang (2023)
*H2AX*	Histone protein; DNA damage repair, chromatin remodeling	Podhorecka et al. (2010)
*HIF1*	Transcriptional factor; regulation of cellular response to hypoxia, angiogenesis, lipid homeostasis.	Shen and Li (2017)

## 1 Advances in estimating individual brain aging trajectory

Molecular processes, despite being related to the same signs of aging, exhibit both linear and nonlinear trajectories throughout life. This is supported by transcriptomic studies of human and rodent tissues at different ages (Greenig et al., 2020; Khan et al., 2017). Recently, using “omics” technologies an ultra-predictive aging clock was developed. This model investigated various factors in the aging process, including sex and health status (Shokhirev and Johnson, 2021). Most research has traditionally modeled aging as a static linear process, failing to capture its dynamic nature (Lehallier et al., 2019). Evidence supporting this comes from transcriptomic studies of human and rodent tissues across various ages. In the human cortex and hippocampus, there is some indication that gene co-expression undergoes exponential changes between the sixth and seventh decades of life. This involves increases in immune-related gene expression and decreases in the expression of certain synaptic genes, such *DNM1, SYN1, SYN2.* For example, mutations in the *DNM1* gene can contribute to neurological dysfunction, including ataxia-like symptoms, due to its critical role in synaptic vesicle recycling and membrane trafficking in neurons. Using a forward genetics approach, it was shown that mutant mice exhibit spontaneous limbic and generalized tonic–clonic seizures, which is due to a spontaneous mutation in the gene encoding dynamin-1. Homozygous mice exhibit a more severe neurologic phenotype at a much earlier age of 3 weeks, including ataxia, hearing and visual defects, and fatal seizures (Matsubara et al., 2024). Mutations in the *SYN1* and *SYN2* genes can lead to epileptic seizures, the development of autistic-like phenotypes, and behavioral changes (Chai et al., 2021; Michetti et al., 2022).

Specifically, when assessing gene expression heterogeneity among individuals across different datasets and brain regions, non-linear changes are observed during middle age. Around age 60, there are exponential increases in gene expression related to axon guidance and mTOR signaling—pathways strongly associated with longevity (Lipton and Sahin, 2014). A similar study of the human hippocampus found an exponential increase in the expression heterogeneity of neurotransmission- and immune-related genes during the transition from the sixth to seventh decade (Dohm-Hansen et al., 2024). Such divergence may reflect differences in individual aging trajectories during middle age, with some individuals aging more rapidly than others. Although the causes of gene expression variability remain to be fully elucidated, the rapidly increasing variance after age 60 (and as early as age 40) has been consistently replicated using various methods and technologies (Dohm-Hansen et al., 2024).

Given recent findings that highlight the diverse early signs of aging and fluctuations in plasma protein levels during middle age, the study of transcriptome changes in blood samples is reasonable (Shen et al., 2022). Analysis of gene co-expression networks provides evidence supporting a decline in synaptic function and an increase in immune activity during brain aging. Transcriptome profiling of brain samples from individuals across different age groups offers valuable insights into the aging process by revealing overall changes in gene expression patterns over time. Expression alterations primarily exhibit temporal dynamics while retaining their spatial specificity. Across various brain regions, age-related changes in gene expression involve decreased synaptic function alongside heightened immune responses (Ham and Lee, 2020).

In the field of predicting individual aging trajectory, comprehensive genomic profiling is increasingly available and has changed the landscape of knowledge about brain aging by providing biomarker information. However, there are many other genes that may also contribute to the definition of accelerated aging. For personalized medicine in the future, deep phenotyping is needed (Katsoulakis et al., 2024). Deep phenotyping is defined as an accurate and comprehensive analysis of phenotypic abnormalities in which a phenotype is observed and includes genetic, epigenetic, clinical, electronic medical and biomedical data, which will lead to a personalized approach in aging. Recent advances in high-throughput omics technologies have facilitated the development of tools to quantify biological aging. These include epigenomic, transcriptomic, and proteomic data that can be integrated with machine learning to create “aging clocks” that identify novel biomarkers of aging (Azzam et al., 2025).

The ability to accurately quantify biological age can help monitor and manage healthy aging. Methylation of CpG subgroups in DNA can be used as a predictor of age. An important milestone in age-related DNA methylation studies has been the development and application of so-called “DNA methylation clocks,” often determined using supervised machine learning methods applied to large cohorts constructed from data from one or more tissues (Horvath and Raj, 2018; Bell et al., 2019). DNA methylation clocks are remarkably robust and in many cases outperform other traditional predictors of biological aging (Horvath and Raj, 2018; Jeong et al., 2024). Epigenetic predictors of aging have broad applications in the study of human health and medicine.

Epigenetic clocks have become a promising tool for estimating biological age, but they have been developed from heterogeneous bulk tissues and thus represent a composite of two aging processes, one reflecting changes in the composition of cell types with age and the other reflecting aging of individual cell types. Epigenomic studies are beginning to reveal substantial heterogeneity in cellular epigenetic programs. Age-associated CpGs have been shown in studies to be highly specific to different cell types. CpGs that vary widely between cell types are particularly susceptible to age-associated DNA methylation changes, leading to divergent epigenetic identities of cell types as humans age (Hannum et al., 2013; Jeong et al., 2024).

Thus, there is a need to dissect and quantify these two components of the epigenetic clock, and to develop an epigenetic clock that can provide an estimate of biological age with cell type resolution. In a recent study, it was demonstrated that in blood and brain, approximately 39 and 12% of the accuracy of the epigenetic clock is due to shifts in lymphocyte and neuronal subsets, respectively. Using brain tissue as a prototype, a neuron-specific DNA methylation clock was created and validated. This cell type-specific clock is shown to provide improved estimates of chronological age in the respective cell and tissue types. Neuron- and glia-specific clocks are found to show an acceleration of biological age in Alzheimer’s disease, with this effect being strongest for glia in the temporal lobe. Moreover, CpGs from this clock show a small but significant overlap with the causal DamAge clock, the damaging clock, which contains only the damaging CpG sites, mapping to key genes associated with neurodegeneration. In contrast, the non-cell-type clock does not show acceleration of biological age or does so only slightly. Thus, there is an importance of studying epigenetic clocks and quantifying biological age with cell type resolution (Ying et al., 2024).

At the cellular level, cross-sectional studies involving large populations have demonstrated that numerous immune components undergo age-related changes (Hotamisligil, 2017; Pawelec et al., 2020). These changes affect both the innate and adaptive branches of the immune system and involve shifts in cellular number and functional capabilities. Although the general decline in immune responsiveness associated with aging, there is typically a moderate increase in circulating inflammatory mediators (IL-1β, IL-6, IL-18, TNF-*α*, etc.), a phenomenon known as “inflammaging.” This chronic, low-grade inflammation is closely linked to many age-related diseases and is a key factor in the reduced cellular responsiveness observed in older adults (Dohm-Hansen et al., 2024). However, aging does not impact all components of the immune system uniformly. Overall, genetic and environmental differences lead to significant variability in immune features among individuals, and this variability increases with age. Previous research has leveraged this variability to identify biomarkers based on individual immune phenotypes at baseline, which are predictive of clinical outcomes. A recent cross-sectional study revealed that healthy human immune states form a continuum rather than distinct categories, with the primary axis of variation driven by features of immune senescence. This indicates that individuals of the same chronological age can exhibit different immune ages. This decoupling of chronological and biological age (Box 1) is also observed in other metrics, including molecular markers like DNA methylation and clinical measures such as frailty, which is considered a form of accelerated aging (Alpert et al., 2019).

This significant inter-individual variability underscores the importance of longitudinal tracking to understand the gradual changes the immune system undergoes with aging. However, existing longitudinal studies on the immune system have typically been either short in duration (weeks to months) or have provided low-resolution data, capturing only a small fraction of the system’s dynamics. The relative stability of the immune system over short periods can create the false impression that immune profiles remain unchanged, whereas longer tracking durations are necessary for systematic longitudinal characterization. Additionally, the immune system’s complexity and variability indicate that data from separate studies focusing on different immune components cannot be seamlessly integrated to form a comprehensive understanding of immunological aging (Alpert et al., 2019). Therefore, a longitudinal study to monitor inflammation throughout life, starting at least from middle age, would be relevant for future research. Additionally, it may be beneficial to identify the pool of genes required for estimating individual brain aging trajectory, considering factors such as individual life history and the presence of diseases (e.g., chronic infections, metabolic disorders, etc.).

## 2 Integrating immunological and metabolic aging

Understanding and modeling the biochemical processes underlying aging could aid in developing predictive models to help clinicians identify individuals at high risk (Zheng et al., 2020). While chronological age is a significant risk factor for many diseases, it does not account for the variability in aging among individuals of the same age. This variability is influenced by individual genetic, biochemical traits, and lifestyle factors (Hertel et al., 2016). The discrepancy between predicted “omic” age and chronological age is utilized to evaluate “accelerated biological age.” Associations between age acceleration and mortality, as well as aging phenotypes, highlight the effectiveness of the clock approach in assessing biological age (Robinson and Lau, 2023). Thus, it is necessary to take into account a sufficient number of factors to determine an individual aging trajectory. Chronic low-grade *inflammation* and altered *metabolic signaling* pathways have been identified as two key themes in the aging process (Chen and Yung, 2019). Furthermore, these subjects are tightly linked under the common idea of meta-inflammation or metaflammation. It’s characterized by a high prevalence of general factors such as IL-6 and TNF-*α* and the occurrence of vicious cycle where the systemic inflammation triggers the metabolic dysfunction and vice versa (Khilazheva et al., 2023; Qu et al., 2022).

Immunological aging is characterized by a decline in immune function (termed “immunosenescence”) and persistent, nonspecific inflammation (termed “inflammaging”) (Franceschi et al., 2018; Komleva et al., 2021). These processes contribute to age-related diseases and syndromes, including infections, cancer, autoimmune disorders, and neurodegenerative disorders, which pose significant health challenges for the elderly worldwide (Guo et al., 2020). Chronic inflammation not only accelerates the aging of the immune system, but also plays a role in various age-related conditions such as Alzheimer’s disease, frailty and cardiovascular diseases (Ferrucci and Fabbri, 2018). Although immunosenescence has been a central focus in studies of immunity and aging, it has faced criticism for lacking universal biomarkers, a clear causal relationship with organismal aging, and its connection to inflammaging (Pawelec et al., 2020; Xu et al., 2020). Over the past decade, significant advancements have reshaped our understanding of immunological aging, revealing new and previously under-appreciated functions and phenomena within the immune system that influence the aging process (Sharma et al., 2022).

Moreover, previous researches suggest a bidirectional relationship between endocrino senescence and the immune system, where declining hormone levels may elevate cytokines such as IL-6 and TNF-α (Straub et al., 2000). Conversely, increased levels of TNF-α and IL-1β near endocrine glands may suppress hormone production and secretion (Eggert-Kruse et al., 2007). Thereby, endocrine dysfunction may contribute to the development of geriatric syndrome frailty through dysregulation of glucocorticoid secretion, insulin-like growth factor signaling, androgen production, and insulin resistance (Sugimoto et al., 2022).

Metabolic aging involves significant changes in the body’s metabolic processes over time. As individuals age, there is a decline in metabolic functions, which results in nutrient sensitivity and an increase in active oxygen forms (reactive oxygen species, ROS) and mitochondrial dysfunction (Guo et al., 2022; Newgard and Pessin, 2014). This includes a reduction in metabolic rate, alterations in energy production pathways, and an increased tendency towards metabolic disorders such as insulin resistance and dyslipidemia. Additionally, with age, the efficiency of cellular processes like glycolysis and oxidative phosphorylation (OXPHOS) declines, leading to a decrease in overall energy production (Lesnefsky and Hoppel, 2006).

Moreover, aging affects the metabolism of different parts of the brain. Aging-related biochemical changes in the brain affect both interregional communication and local metabolic processes. Alterations in white matter integrity disrupt interactions between the prefrontal cortex and key structures such as the hippocampus and striatum, leading to impairments in tasks that rely on processing speed and memory retention, both immediate and delay. Additionally, the brain experiences a progressive decline in energy metabolism over time (Camandola and Mattson, 2017). A global important change is the decrease in brain glucose consumption, which leads to a decrease in the degree of aerobic glycolysis in different parts of the brain (Goyal et al., 2017). Functional neuroimaging studies indicate that glucose hypometabolism and mitochondrial dysfunction are among the earliest detectable functional alterations associated with normal brain aging. Research in both humans and animal models has demonstrated an age-related reduction in the expression of glucose transporters in the brain (Ding et al., 2013), along with significant modifications in the expression of enzymes essential for glycolysis and oxidative phosphorylation. In aging mice, ATP levels in white matter are diminished, coinciding with ultrastructural changes in mitochondria and a weakened association between mitochondria and the endoplasmic reticulum (Stahon et al., 2016). With the course of aging of the body, a decrease in hexokinase activity, citrate synthase activity, malate dehydrogenase activity, glutamate dehydrogenase activity, glutamate-pyruvate transaminase activity and acetylcholinesterase activity could be observed (Villa et al., 2013).

Measuring biological age through metabolomic analysis, which captures individual variability, could serve as a valuable predictor with numerous practical applications in personalized medicine. These applications include distinguishing between accelerated and delayed biological aging (Hertel et al., 2016). Metabolic changes involving advanced glycation end products (AGEs) are linked to inflammatory responses and may contribute to accelerated aging (Table 2). The Rotterdam Study found that lower levels of the soluble receptor for advanced glycation end products (sRAGE) were correlated with a higher prevalence of dementia, though this association was not observed with the incidence of dementia (Fang et al., 2022). Proper activation of the RAGE-ligand axis results in a cellular phenotype where the transcription factor NF-κB is activated, leading to the overproduction of pro-inflammatory mediators (IL-1, IL-6, IL-18, TNF-*α*, CXCL10). Variations in the genes encoding cytokines have been linked to longevity. A previous study by Balistreri et al. found that male centenarians often possess allelic variants that suppress inflammation, suggesting they are better equipped to defend against major age-related disorders (Balistreri et al., 2012; Falcone et al., 2013). These findings highlight the intricate relationship between genetic factors of metaflammation in aging.

**Table 2:** The current researches of potential hallmarks of accelerated brain aging.

Biomarker	Years	Researchers	Results	Reference
Mitochondrial impairments markers researches				
Drp1 and Mfn1/2	2020	Zhang, X. et al.	Connection between overexpression of *Drp1* and NLRP3 activation in OLs (oligodendrocytes) with inflammation and axon degenerations with the high level of gene expression of *Drp1*, *Nlrp3* and IL-1β in patients with AD and transgenic 5xFAD mice. Approximately the same results in cell line of OLs with Aβ(1–42)-oligomer therapy. Mfn1/2 alterations were not detected. OL-specific heterozygous knockout of *Drp1* correlated to decreasing of NLRP3-associated inflammation level, renewal of cognitive function and reducing of axons and myelin damages in mice model of AD as a consequence.	Zhang X. et al. (2020)
	2022	Mishra, E. et al.	Morphological alterations such as the mitochondrial membrane’ damages and mitochondrial fragmentation and shape change in aged mice. Gene expression changes associated with apoptosis proteins (Bcl-XL), mitochondrial dynamic (Drp1, Mfn1/2) and memory (Arc). Memory loss (as well as decreased expression of the Arc-protein), reduction of fusion proteins Mfn1/2 and Bcl-XL, and an increase in the number of fission proteins.	Mishra and Thakur (2022)
Neuroinflammation markers researches				
NLRP3	2020	Liu, Y. et al.	An raising of *Nlrp3* gene expression level in Aβ(1–42) oligomer-induced BV-2 microglia cell line in comparison to the normal BV-2. Application of TLR4-inhibitor CLI-095 was found to reduce the NLRP3 expression in Aβ(1–42) oligomer-induced BV-2 and also in primary microglia.	Liu Y. et al. (2020)
	2022	Vizuete, A. et al.	An increase in mRNA of NLRP3 (mRNA/β-actin) was detected in the lipopolysaccharide (LPS)-induced inflammation rats’ model (*in vivo*) after 6 h. Increased *Nlrp3* (mRNA/β-actin) gene expression in LPS-induced neuroinflammation in acute hippocampal slices (ex vivo).	Vizuete et al. (2022)
	2023	Khilazheva, E. et al.	Increased level of NLRP3, HMGB1 and IL-18 expression in WT-14-month-old mice; No increase of SASP cells in NLRP3 knockout mice during aging	Khilazheva et al. (2023)
IL-6	2021	Zeng, L. et al.	An evaluation of IL-6 in the brain tissues in a rat model of D-galactose-induced neuroinflammation MOD group In the L-theanine treatment groups with different doses of L-theanine, there was a decrease in IL-6 levels.	Zeng et al. (2021)
	2021	Lyra e Silva, N. et al.	Increased levels of IL-6 have been shown in the post-mortem cingulate cortex and plasma of Alzheimer’s patients compared to age-matched control brains, leading to cognitive and metabolic impairment.	Lyra e Silva et al. (2021)
RAGE	2021	Walke, P. et al.	A possible cause of insulin resistance in a cell culture of CHO-IR-GLUT4 induced by glycated insulin (GI) which affects glucose uptake by cells. The use of glycated insulin increases the level of oxidative stress in cells CHO-IR-GLUT4 and also RAGE expression depending on the concentration of GI as opposed to nonmodified insulin.	Walke et al. (2021)
	2021	Zeng, L. et al.	Increasing of RAGE gene expression and protein expression were observed in the D-galactose-induced neuroinflammation model (positive control). The study also demonstrated the impact of L-theanine on the expression of RAGE proteins and genes. A notable reduction in RAGE quantity was observed as the concentration of the treatment increased.	Zeng et al. (2021)
	2022	Vizuete, A. et al.	In vivo experiment on the lipopolysaccharide (LPS)-induced neuroinflammation rats’ model has not showed the variation of mRNA of RAGE after 6 h and 24 h after inflammation. Ex vivo experiment in acute hippocampal slices after incubation with LPS for 1 h revealed a considerable rise in RAGE gene expression and its protein expression.	Vizuete et al. (2022)
p16/p21	2020	Wei-Hsiang Hsu et al.	On the model of human neuroblastoma SH-SY5Y with D-gal (or different concentrations of beryllium salts), there were increased numbers of aging markers such as p16 and p21 as well as the SA-β-gal activity. The effect of T1-11 (an adenosine analog) on adenosine receptor A2AR activation was investigated. It was suggested that A2AR activity would reduce cellular senescence, which was confirmed by a decrease in p16, p21 and SA-β-gal levels in SH-SY5Y with higher concentration of T1-11.	Hsu et al. (2020)
	2021	Qian, J. et al.	The researchers identified a rise in senescent molecules, namely p16 and p21, in hippocampal tissues in the group that had been injected with D-galactose than in the control group. This was evidenced by an increase in mRNA and protein levels. Both puerarin and dihydromyricetin (DMY) have been observed to reduce the protein and gene expression of p16 and p21. The impact of DMY on the group of mice with D-gal is more significant.	Qian et al. (2021)
	2022	Torres, P. et al.	It was discovered that elevated profile of p16 was noted with progression of the disease, while p21 gene expression increased only in the final stage in the hSOD1-G93A mouse model. It has been demonstrated that the senolytic treatment, such as Navitoclax, is not affected by the cellular senescence molecules. In contrast, the dasatinib–quercetin combination has been demonstrated to positively affect the downregulation of p16 and p21.	Torres et al. (2022)
Cell competition markers researches				
Myc / c-Myc	2013	Greer, C. et al.	The overexpression of *Myc*-gene in Drosophila leads to increased number of the accidental DNA double-strand breaks (DSBs), counting with LacZ reporter gene, that result in genomic instability. Moreover, it was shown that *Myc*-hyperexpression reduces the Drosophila lifespan almost in half while the heterozygous flies have got the lower mutation rate and altered longevity.	Greer et al. (2013)
	2021	Chen, Y. et al.	The positive influence of dexmedetomidine (DEX) was determined in the (LPS)-induced model of neuroinflammation. It had been demonstrated the reverse action of neuron damages caused by LPS in the rats’ hippocampus, also as minimization differences in proinflammatory cytokine levels (IL-1β, IL-6, IL-18, and TNF-*α*). It was confirmed the inhibitory activity of DEX on c-Myc activation and c-Myc/chloride intracellular channel 4 (CLIC4) pathway which stimulate apoptosis of hippocampal neurons due to stress factors.	Chen et al. (2021)
JNK	2019	Khan, M. et al.	Neuroprotective effects of anthocyanins investigated in (LPS)-induced model of neuroinflammation in C57BL/6 N mouse involving JNK/Akt/GSK3β signaling pathway. It was described the high level of p-JNK in hippocampus of adult mice (8 weeks old) also as in HT22 cell line. The impact by anthocyanin showed considerable reduction of p-JNK in cells and tissue. In addition, anthocyanin turned down the rates of markers of apoptosis (Bax/Bcl2, cyt-C) and inflammation (NF-κB, TNF-α и IL-1β). The activity of the anthocyanin was homologous to a specific JNK inhibitor.	Khan et al. (2019)
	2020	Ibrahim, W. et al.	The same results were demonstrated in the model of D-galactose (D-gal)-exposed Wistar rats as AD model. Diapocynin (or Diapo) treatment - NOX inhibitor – had a direct impact on JNK/c-Jun pro-apoptotic cascade and normalization of p-tau level.	Ibrahim et al. (2020)

It has long been established that a lack of physical activity, a sedentary lifestyle, mental health issues, an excess of calories and alcohol abuse can result in a poor metabolic rate (Kullmann et al., 2016). In addition, it is essential to highlight that another adverse effect is the reduction or disruption of glucose flow to the brain (Komleva et al., 2021). Accordingly, the normally functioning of brain cells is dependent upon insulin signaling. The regular activation of serine/threonine kinase AKT (via the insulin–IRS 1/2–PI3K–AKT signaling cascade) is responsible for mediating glucose transport by facilitating the translocation of GLUT4 to the membrane, as well as for activating mTOR, blocking GSK-3*β*, and regulating FoxO factors (Akintola and van Heemst, 2015; Pomerantz et al., 1997; van der Heide et al., 2006; Yin et al., 2013). Therefore, the preservation of insulin sensitivity in the human body represents a critical factor in achieving longevity. A number of studies have demonstrated that brain insulin resistance associated with a reduction in insulin and insulin-like growth factor 1 (IGF-1), inactivation of the insulin receptor substrate 1/2 (IRS 1/2), Akt and mTOR and GSK-3β phosphorylation (Hölscher, 2020; Schubert et al., 2004; Sedzikowska and Szablewski, 2021). Changes in GSK3 expression and function are strongly associated with age-related diseases (Souder and Anderson, 2019). Consequently, there is impairment of the insulin/IGF-1 signaling (IIS pathway), hyperphosphorylation of the Tau protein, and an increase in pro-inflammatory cytokines (IL-1β, IL-6, TNF-α, TGF-β) (Akintola and van Heemst, 2015; Maciejczyk et al., 2019). These processes have been shown to adversely affect a number of cellular functions, including proliferation, the cell cycle, synaptic activity, neurogenesis, mitochondrial activity, the ability to counter oxidative stress, memory formation, and neurocognitive deficits (Wang et al., 2022).

Understanding and modeling the biochemical processes underlying aging can aid in developing predictive models to help clinicians identify individuals at high risk. While chronological age is a significant risk factor for many diseases, individual genetic, biochemical traits, and lifestyle factors contribute to variability in aging. Measuring biological age through metabonomic analysis, which captures this individual variability, could serve as a valuable predictor with numerous practical applications in personalized medicine, including distinguishing between accelerated and delayed biological aging. Addressing brain insulin resistance and maintaining metabolic health are crucial for mitigating the effects of aging and improving overall health outcomes.

## 3 Genetic susceptibility to impaired brain bioenergetics

Metabolic impairment along with alterations in nutrient availability leads to *mitochondrial dysfunction* and can influence on the ROS rate or oxidative phosphorylation potency. Recent studies have highlighted the critical role of OXPHOS in brain aging, emphasizing the involvement of both mitochondrial and nuclear genes in regulating this essential energy metabolism process, which is closely linked to longevity (Guo et al., 2022). For instance, the gene *CG11837*, a putative ortholog of the human *DIMT1*, has been shown to regulate lifespan in various species particularly worms and diverse insects (Tao et al., 2024). Knockdown of *CG11837* reduces the median lifespan in diverse insect species and *Caenorhabditis elegans*, while its overexpression extends median lifespans in fruit flies and *C. elegans*, enhancing the activity of OXPHOS genes (Tao et al., 2024).

Moreover, Verma et al. reported that *DIMT1* gene, when silenced in insulin-secreting cells, has been found to impair mitochondrial function significantly. This impairment is characterized by lower expression levels of mitochondrial OXPHOS proteins, a reduced oxygen consumption rate, dissipated mitochondrial membrane potential, and a slower ATP production rate (Verma et al., 2022). Such disruptions in mitochondrial function are crucial as they underline the importance of OXPHOS genes in maintaining cellular energy balance and longevity, particularly in the context of aging.

Another critical gene associated with brain aging is *Dnm1l*, which encodes the protein Drp1. Drp1 is a mechano-chemical GTPase responsible for regulating mitochondrial fission, a process essential for maintaining cell conditioning (Mishra and Thakur, 2022). Dysregulation of Drp1 has been linked to various neurological disorders, including Alzheimer’s, Parkinson’s, and Huntington’s diseases (Lin et al., 2022). Notably, mutations in the human *Drp1* gene can lead to severe neurodevelopmental defects, post-neonatal lethality, developmental delay, late-onset neurological decline, or optic atrophy (Ishihara et al., 2009). A potential relationship to longevity and/or aging has also been shown, namely short-term overexpression of *Drp1* in middle-aged flies reduced the ROS of mitochondria and restored proteostasis processes thereby it is increased mean lifespan of flies (Rana et al., 2017).

Moreover, Drp1 is produced in multiple isoforms through alternative splicing of its mRNAs, with the Drp1_ABCD_ isoform playing a unique role in neuronal function (Itoh et al., 2018). Drp1_ABCD_ is enriched in dendritic spines and regulates postsynaptic clathrin-mediated endocytosis by positioning the endocytic zone at the postsynaptic density, independently of mitochondrial fission (Itoh et al., 2019). Loss of Drp1_ABCD_ leads to the formation of ectopic dendrites in neurons and enhanced sensorimotor gating behavior in mice, indicating its critical role in controlling postsynaptic endocytosis, neuronal morphology, and overall brain function. In hippocampal neurons, the absence of Drp1 or expression of dominant-negative Drp1 does not lead to cell death but results in bioenergetic and synaptic function deficits (Shields et al., 2015). Similarly, *Drp1* knockout (KO) in hypothalamic pro-opiomelanocortin neurons shows that these neurons are viable and exhibit increased glucose and leptin sensing (Divakaruni et al., 2018).

Generally, the regulation of mitochondrial function and dynamics through genes like *DIMT1* and *Drp1* is vital for maintaining neuronal health and function during aging. The observation gained from these studies underscore the complex interplay between genetic factors and mitochondrial dynamics in the aging brain, offering potential therapeutic targets for mitigating age-related neurological disorders. Therefore, understanding mitochondrial function is crucial for assessing individual aging trajectories, particularly in the context of neuronal health.

Other key proteins for mitochondrial dynamics are mitofusins (Mfn1/2) involved in outer mitochondrial membrane fusion events (Ishihara et al., 2009; Paul et al., 2021). Moreover, it was found the Mfn2 protein is expressed precisely in brain and skeletal muscles whereas Mfn1 is a main isoform and expressed mostly in liver and pancreas (Sharma et al., 2019; Hernández-Alvarez and Zorzano, 2021). Some studies have described an increase in ROS production and JNK activity in insulin signaling with Mfn2 expression deficiency in adult mice leading to metabolic alterations and insulin resistance (Sebastián et al., 2012). Other research has confirmed the morphological changes of mitochondria during aging (Mishra and Thakur, 2022). They discovered a correlation between hippocampal mitochondrial morphology, recognition memory function and fission/fusion gene expression. Thus, there were detected fragmented and round shaped mitochondria with a lack of *Mfn1/2* gene expression with recognition memory decline in old mice (80 weeks old) in compared with adult (30 weeks old) and young (10 weeks old) groups (Mishra and Thakur, 2022). Alterations in gene expression with age, probably, can make a strong influence on mitochondrial dynamics, shape and also cristae state.

Mitochondrial dysfunction, in particular imbalance between membrane fission and fusion, as a hallmark of aging, is linked to various neurodegenerative and age-related diseases (Table 2). Given the pivotal role of mitochondria in energy production and cellular metabolism, assessing mitochondrial function provides valuable signs of the aging process. Moreover, recent studies have demonstrated the significance of single nucleotide polymorphisms (SNPs) in the context of aging. For instance, SNPs from four loci, especially at the chromosome 17 locus, have been significantly associated with relative biological age. Within this locus, genes such as *MAPT* or *Wnt3* have noteworthy gene-level associations with relative biological age (Ning et al., 2021). Thus, mutations in the tau-encoding gene *MAPT*, which are associated with a range of neurodegenerative, lead to significant mitochondrial dysfunction (Szabo et al., 2023). Early stages of tauopathy are marked by substantial impairments in mitochondrial function, with mutant tau compromising nearly every aspect of mitochondrial operation. Furthermore, mitochondria play a crucial role in regulating stem cell function as well as perturbations in mitochondrial dynamics are closely associated with cellular senescence (Miwa et al., 2022). A deeper understanding of mitochondrial dysfunction during aging and age-related metabolic diseases is crucial for developing therapies to address late-life morbidities.

## 4 Age-related trends of gene expression in neuroinflammation and brain senescence

There is evidence for independent evaluation the process of brain aging, it is essential to employ an approach that consists of a cognitive test for the purpose of detecting mild cognitive impairment, the *number of senescent cells* (SCs), and the gene expression profile of potential biomarkers. Identification of expression levels of selected markers of aging can be used for detection of significant changes in the organism, such us metabolism and cell cycle disturbance, as well as the presence of chronic inflammation (Baechle et al., 2023). Further research has sought to establish whether a more accurate prediction of brain age can enhance the characterization of brain aging and aid in identifying novel genetic factors.

Recent studies have highlighted the critical role of genes in regulating inflammation and cellular senescence, which are pivotal processes in the trajectory of individual aging. One such study utilizing the APPswe/PS1ΔE9 mouse model, which carries two transgenes for mutant proteins associated with Alzheimer’s disease, demonstrated a premature phenotype of immunosensitivity (Luan et al., 2024). The phenotypic differences observed were primarily due to severe immunologic aging, as indicated by a high proportion of depleted T-lymphocytes in AD mice compared to age-matched wild-type mice (Luan et al., 2024). Furthermore, it has been reiterated that the terms “immune system in aging humans” and “immunosenescence” are sometimes misinterpreted, and this study shows that these two concepts are fundamentally different (Mogilenko et al., 2022; Nikolich-Žugich, 2018). Based on these data, the damage caused by normal aging is not as severe as immunologic aging; immunologic aging is much more dangerous than aging (Luan et al., 2024). This suggests that the damage from immunologic aging surpasses that of normal aging, emphasizing the importance of targeting immunologic aging to increase longevity and refine the trajectory of individual aging.

Using UK Biobank data, it was reported a mean absolute error of about 3.5 years between prediction of brain age and chronological age and identified a significant association with a chromosome 17 locus, including the *MAPT* gene, known for its mutations linked to dementia and Parkinson’s disease and AD (Ning et al., 2021). Moreover, studies from GWAS landscape have identified that *KLF3, KANSL1, MAPT-AS1, CRHR1, NSF, RUNX2*, and *NKX6-2* gene were significantly associated with brain aging (Kim et al., 2023). *KLF3* has been shown to mediate lifespan in *C. elegans* and may be linked to educational attainment in humans (Hsieh et al., 2017). Additionally, long non-coding RNA (lncRNA) KLF3-AS1 plays a critical role in exosomal NLRP3 regulation (Li et al., 2022). This positions *KLF3* as a fundamental suppressor of NF-κB–driven inflammation (Leonardsen et al., 2023). The *KANSL1* gene plays the role for cognition (Arbogast et al., 2017). Other notable genes include *RUNX2*, associated with osteocalcin expression and cognitive function decline, and *NKX6-2*, which regulates myelin sheath formation and maintenance in the central nervous system (Nakamura et al., 2021; Southwood et al., 2004). Also, studies have shown that *RUNX2* activation is reduced by IL-1*β* and through activation of the MAPK pathway, and knockdown of NLRP3 notably enhanced the expression of *RUNX2* (Jiang et al., 2021).

The integration of the genetic insights with measures of biological age, inflammation, and mitochondrial function could significantly advance our understanding of individual aging trajectories and support the development of targeted interventions to mitigate aging-related diseases and conditions (Ning et al., 2021).

Another important aspect of the accelerated aging process is the increase and accumulation of the number of senescent cells. Senescent cells are cells that have ceased dividing and entered a state of irreversible cell cycle arrest in response to various stressors. These can be external aggressors such as X-irradiation, UV light, and therapeutic interventions, or internal factors such as DNA damage, chromatin disruption, oxidative stress, and telomere shortening (Baker and Petersen, 2018; Coppé et al., 2010; Cox, 2022). This type of cell exhibits a distinctive phenotype, which includes the overexpression of cyclin-dependent kinase (CDK) inhibitors p21^Cip1^ and p16^INK4a^, an increased activity of a β-galactosidase pH 6,0 (SA-β-gal), and the secretion of SASP (Liu, 2022; Sacco et al., 2021; Zhang et al., 2019). The permanent expression of p21 and p16 inhibits the activity of CDK2, CDK4, and CDK6, resulting in the retinoblastoma protein being in a hypophosphorylated state (Baker and Petersen, 2018; Childs et al., 2017; Prieto and Baker, 2019). This suppresses gene expression in the S phase of the cell cycle. In addition, senescent cells secrete the *senescence-associated secretory phenotype* (SASP) which includes the proinflammatory biomolecules such as interleukin (IL)-1β, IL-6, IL-8, and chemokines (CCL-2, CXCL-12), growth factors, TNF-*α*, metalloproteinases (MMPs), etc. (Baker and Petersen, 2018; Sacco et al., 2021).

It is a common phenomenon for neuroinflammation also as for metabolic imbalance or bioenergetics impairments produce circulating inflammatory molecules that induce a low-grade chronic inflammation. Inflammation triggers heightened permeability of the vascular blood–brain barrier to cytokines and chemokines, thereby disrupting neuronal and glial homeostasis and perturbing brain equilibrium (Galea, 2021). A growing body of research correlates heightened inflammation with alterations in brain structure and function, cognitive impairment, and elevated susceptibility to dementia (Hotamisligil, 2017; Ogrodnik et al., 2021). Therefore, another potential marker as NLRP3, RAGE, p16/p21, and IL-6 could be detected for evaluation of individual brain aging trajectory (Table 2). The advanced rates of these hallmarks are linked with the permanent activation of M1-type microglia, the accrual of advanced glycation end products, and the increasing number of senescent cells.

Therefore, evaluating brain aging requires a comprehensive approach that includes not just cognitive testing and MRI imaging, but also assessing the dynamic of cell fate, senescence, and analyzing gene expression profiles of potential biomarkers. Recent studies highlight the role of genetic factors, particularly those involved in neuroinflammation and cellular senescence, in determining the trajectory of aging. Understanding cell state and its impact on neighboring ones, as well as their formation of a specific microenvironment, can support the developing interventions to mitigate age-related diseases and cognitive decline.

## 5 Stem cell exhaustion and quiescence in accelerated brain aging

An essential aspect of normal tissue homeostasis and regeneration is a presence of *healthy tissue-specific stem cells* which can save the strong balance between quiescence and proliferation for efficient replacement of ageing or damaged cells (Oh et al., 2014). Distinctive mechanism of adult stem cells is a capability to keep on non-proliferative state that named quiescence for avoidance of DNA mutations during cell division. It is known, that the fraction of cells in a resting state is located primarily in low cellular turnover tissues in brain or heart in contrast to the other type of tissues where the cellular turnover is very high (e.g., epithelial tissue) (Liu and Rando, 2011). Maintaining a quiescence state is necessary for functional capacity preservation of cells and organs. Thus, the imbalance of quiescence and proliferation caused by aging or accumulation of various pathogenic signals from stem cell niche can result in stem cell exhaustion in reserve pool of cells (Andreotti et al., 2019; Urbán and Cheung, 2021).

Moreover, in the context of brain aging, it has been shown that fraction of quiescent neural stem cells (qNSCs) increases over the course of a lifetime whereas neurogenesis in hippocampus (SVZ/SGZ) reduces (Audesse and Webb, 2020; Kalamakis et al., 2019). As a result, qNSCs are aging and take on some resilience for activation and differentiation, thereby they are incapable to recover of brain damages and age-related cognitive decline (Kalamakis et al., 2019). However, the most interesting outcome is that aged NSCs and young NSCs after activation from a resting state may demonstrate similar transcriptomic and functional profile (Kalamakis et al., 2019). Particular importance for the normal NSCs functioning possesses microenvironment condition of a neighboring niches (Navarro Negredo et al., 2020; Schultz and Sinclair, 2016). As mentioned previously, aging stimulates recycling of pro-inflammatory cytokines alongside with increasing rate of reactive nitrogen and oxygen intermediates by M1-microglial cells during low-grade systemic inflammation (Zhang Z. et al., 2020, Wang et al., 2023). Thus, Kalamakis et al. (2019). discovered relation between inflammatory response and fraction of qNSCs, which allowed them to conclude that permanent pro-inflammatory molecules secretion induces the maintenance the stable quiescence state with canonical Wnt-signaling pathway.

In addition to activated microglia, nutrient-sensing signaling and energy metabolism play a critical role in maintaining the balance between quiescence and activation of stem cells. Under normal low-oxygen conditions, qNSCs predominantly utilize glycolytic metabolism rather than mitochondrial oxidative phosphorylation (Cavallucci et al., 2016; Yu et al., 2024). Thus, this is due to hyperexpression of the hypoxia-inducible factors (HIFs, particularly HIF-1) that are activated under oxygen-deprived state and contribute the stem cell pool maintenance and their resting state (Cavallucci et al., 2016). Accordingly, the metabolic changes (such as destruction of HIFs in excess of oxygen) may promote the switch of NCSs from state of rest to an active one (Urbán and Cheung, 2021). Closely related to energy functions, nutrients sensitivity is also modified in different stem cell states. One of the main metabolic pathways that response for nutritional input and longevity is insulin/insulin-like growth factor (IGF) signaling (IIS) causes FoxOs transcription factors nuclear export and further ubiquitination (Renault et al., 2009; Tatar et al., 2003). Renault et al. (2009) were determined that total inhibition FoxOs (especially FoxO3) with PI3K/Akt proteins of IIS results in excessive differentiation of NCSs to neural lineages and early stem cell exhaustion from the reserve pool. The insulin/IGF-1 signaling (IIS) knockout, specifically IGF-1R knockout (IGF-1R KO), leads to reductions in neurogenesis and cell self-renewal within the neurogenic niche\cite (Chaker et al., 2016). However, cyclic variations in IGF-1 levels, achieved through modifications such as prolonged fasting or normal dietary patterns, have been shown to improve cognitive performance and hippocampal neurogenesis in aged mice (Brandhorst et al., 2015). Additionally, other components of the nutrient-sensing signaling pathways downstream of IIS, such as PI3K/mTOR, play a crucial role in maintaining stem cell balance. The suppression of the mTOR pathway contributes to the preservation of the quiescent state of stem cells (Navarro Negredo et al., 2020). Conversely, sustained high activity of mTOR is linked to the reactivation of cells, which can lead to their exhaustion within the reserve cell pool (Audesse and Webb, 2020).

Thus, knowledge and understanding of the processes of the maintenance of healthy neural stem cell pool in hippocampal neurogenic niches is necessary for biological brain aging due to the fact that it may be effective for decrease the risk of neurodegeneration and also for keeping the normal neurogenesis and cognitive functions during the individual aging trajectory development (Navarro Negredo et al., 2020). Moreover, it is important to realize the relationship between quiescent and active cells ant the impact of internal and external triggers on this.

## 6 Cell competition and senescence in a strategy for estimating brain aging

**Figure 2:**
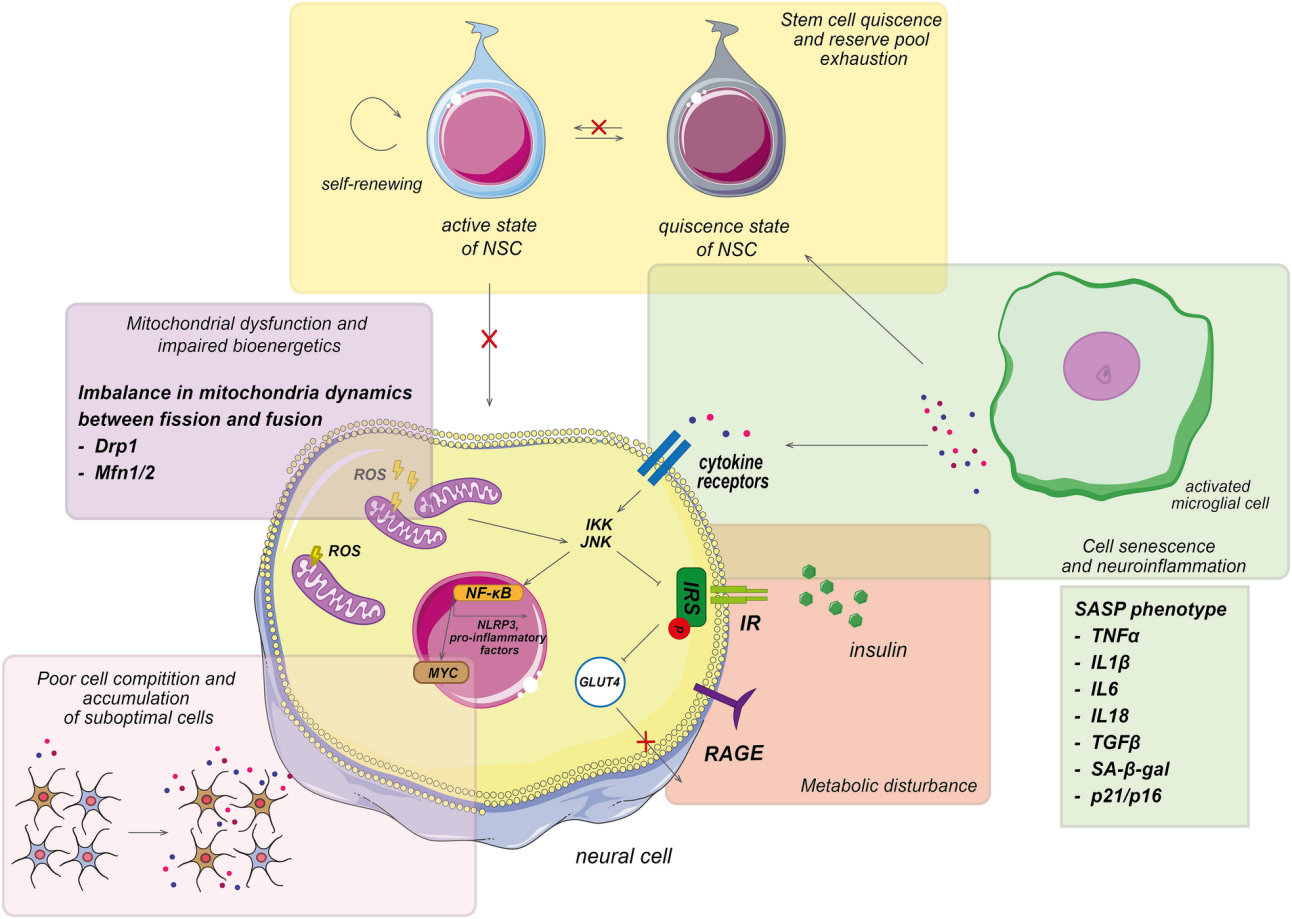
Pathological processes involved in aging trajectory. Main pathological processes associated with aging, which characterized by the accumulation of senescent cells, mitochondrial dysfunction, impaired biogenesis, cellular senescence, poor cell competition, and metabolic disturbance. Which display a senescence-associated secretory phenotype (SASP) and exhibit normal cell cycle inhibition. The accumulation of the senescent cells leads to an increase in pro-inflammatory factors, which affect neighboring cells. The interaction of pro-inflammatory agents with cellular receptors activates various JNK-involved signaling pathways, resulting in metabolic impairment, increased reactive oxygen species (ROS) formation, and further elevation of pro-inflammatory molecules, ultimately contributing to systemic inflammation. Additionally, the SASP phenotype inhibits the differentiation and proliferation of active stem cells, disrupting normal cell formation. Consequently, senescent cells dominate cellular competition, and their elimination is impeded, exacerbating the aging process. NSCs, neural stem cells.

In the context of brain aging, the role of genes, that regulate cell behavior, particularly through cellular competition, has been highlighted. We aim to clarify the role of *cell competition* in the cellular selection processes that are known to influence the lifespan of model organisms (Tano et al., 2019; Yeates et al., 2023). Cell competition is tightly connected to the processes of brain aging outlined above (Figure 2). In this section, the immunosenescence and inflammation are discussed—one of the hallmarks of aging—within the context of cell competition to understand individual aging trajectories.

It is well-established that aging leads to changes that reduce the system’s and the organism’s overall resilience to various stresses (Merchant et al., 2022). This reduction in adaptability fosters changes that contribute to the development of age-related diseases and geriatric syndromes such as frailty, characterized by decreased systemic robustness (Ferrucci and Fabbri, 2018). Analogous to the entire system, cells also experience a decline in resilience with age. According to the mechanism of cellular competition, non-functional unfit cells are gradually eliminated (Bowling et al., 2019; Merino et al., 2016). However, aging inhibits cellular competition, resulting in the accumulation of suboptimal cells, which in turn reduces lifespan contributing accelerated aging (Marques-Reis and Moreno, 2021). Alongside this, cellular senescence becomes prominent during aging. Therefore, these two interconnected processes—cellular competition and cellular senescence—play crucial roles in aging. In connection with the immune system weakens, it cannot eliminate senescent cells, which then adopt a senescent phenotype, factors that negatively impact neighboring cells and exacerbating the aging process (Marques-Reis and Moreno, 2021). Some specific genes regulate the delicate balance between cell competition and cellular senescence, influencing aging trajectories (Bondar and Medzhitov, 2010; Marques-Reis and Moreno, 2021). Herewith, in accelerated aging, a disruption in this balance results in increased senescent cell accumulation and diminished cell competition. In contrast, in delayed aging, robust cell competition processes prevail, limiting cellular senescence. This underscores the importance of understanding the mechanisms behind cell competition and senescence, as well as their implications for aging and disease management.

Cell competition occurs in adult nervous tissues and crucial for maintaining adult tissue health, and delaying aging (Coelho and Moreno, 2019; Merino et al., 2016). This process operates through three non-exclusive modes: competition for extracellular survival factors, fitness fingerprints, and mechanical stress (Costa-Rodrigues et al., 2021). Cell competition involves heterogeneous fitness levels within a tissue, where “loser” cells are eliminated through various mechanisms, including apoptosis, extrusion, senescence, phagocytosis, or differentiation (Bowling et al., 2019; Costa-Rodrigues et al., 2021). Interestingly, even wild-type cells can be eliminated in a process called “super-competition” (Coelho and Moreno, 2019). For example, overexpression of the transcription factor Myc can convert “loser” cells into “winners,” outcompeting wild-type cells. Several mutations, affecting pathways such as p53, JAK/STAT, Hippo, and WNT, are known to induce super-competition (Bowling et al., 2019; Coelho and Moreno, 2019; Costa-Rodrigues et al., 2021). Overall, cell competition acts as a vital quality control mechanism, ensuring the removal of aberrant cells and maintaining tissue health throughout the organism’s life.

Recent studies have identified the Toll/NF-κB pathway, a component of the innate immune response, as crucial for recognizing and eliminating “loser” cells (Germani et al., 2018). Further research is needed to explore this pathway’s role in other competition scenarios and its potential links with the fitness fingerprint pathway (Germani et al., 2018). Additionally, circulating macrophages play a critical role in removing cellular debris post-apoptosis. The chemoattraction of hemocytes to dying loser cells is regulated by the secretion of tyrosyl-tRNA synthetase (TyrRS), driven by JNK activation in “loser” cells (Li et al., 2020).

Furthermore, a recent study examined the molecular mechanisms underlying neuroepithelial cell competition, with a particular focus on the elimination of juvenescence-losing cells from the neuroepithelium (Jam et al., 2020). The findings underscore the role of cell competition as a critical quality-control mechanism orchestrating brain homeostasis. Myc-driven cell competition in the mouse epiblast revealed a decrease in bone morphogenetic proteins (e.g., Srsf7 and Ezh2) signaling specifically in “loser” cells (Jam et al., 2020). Notably, it was observed that neuroepithelial cell competition involves the acute loss of Srsf7 (Kadota et al., 2020) and Ezh2 (Tano et al., 2019) in loser cells prior to their elimination, suggesting these factors play pivotal roles in maintaining cellular juvenescence. These results provide evidence that senescent cells losing cellular juvenescence may trigger cell competition, leading to their selective removal from the neuroepithelium through apoptosis (Jam et al., 2020). Thus, suppression of Srsf7 resulted in the loss of cellular juvenescence, mediated by Ezh2 suppression at both mRNA and protein levels. This loss was further evidenced by increased levels of proteins P16 and gamma H2AX associated with senescence (Jam et al., 2020).

Additionally, it was known, increased H3K27me3 levels are basic epigenetic characteristic of aging and exhibit a distinct genomic pattern in aged tissues, featuring loss of peaks and gain of age-domains (Yang et al., 2023). This type of chromatin modification directly associated with catalytic component of polycomb repressive complex 2 (PRC2) Ezh2. The decline in Ezh2 expression with age the cell to integrate different signals and decide between survival or apoptosis (Jam et al., 2020).

More examples of pathways were found for understanding their role in apoptosis is important for the development of novel therapeutic strategies. Among others, JNK and p38 MAPK inhibitors can be used to treat inflammatory and neurodegenerative diseases, whereas ERK activation may be useful to target cancer cells (Costa-Rodrigues et al., 2021; Lavoie et al., 2020; Machado et al., 2021). These findings contribute to our understanding of how cell competition and molecular mechanisms involving Srsf7, Ezh2, JNK, ERK, Myc, MAPK, and H3K27me3 regulate cellular senescence and aging processes in the brain. Further research into these pathways could uncover potential targets for interventions aimed at promoting healthy brain aging and mitigating age-related neurodegenerative diseases (Table 2).

## 7 Challenges and potential therapeutic strategies in targeting neuroinflammation and cellular senescence

**Figure 3:**
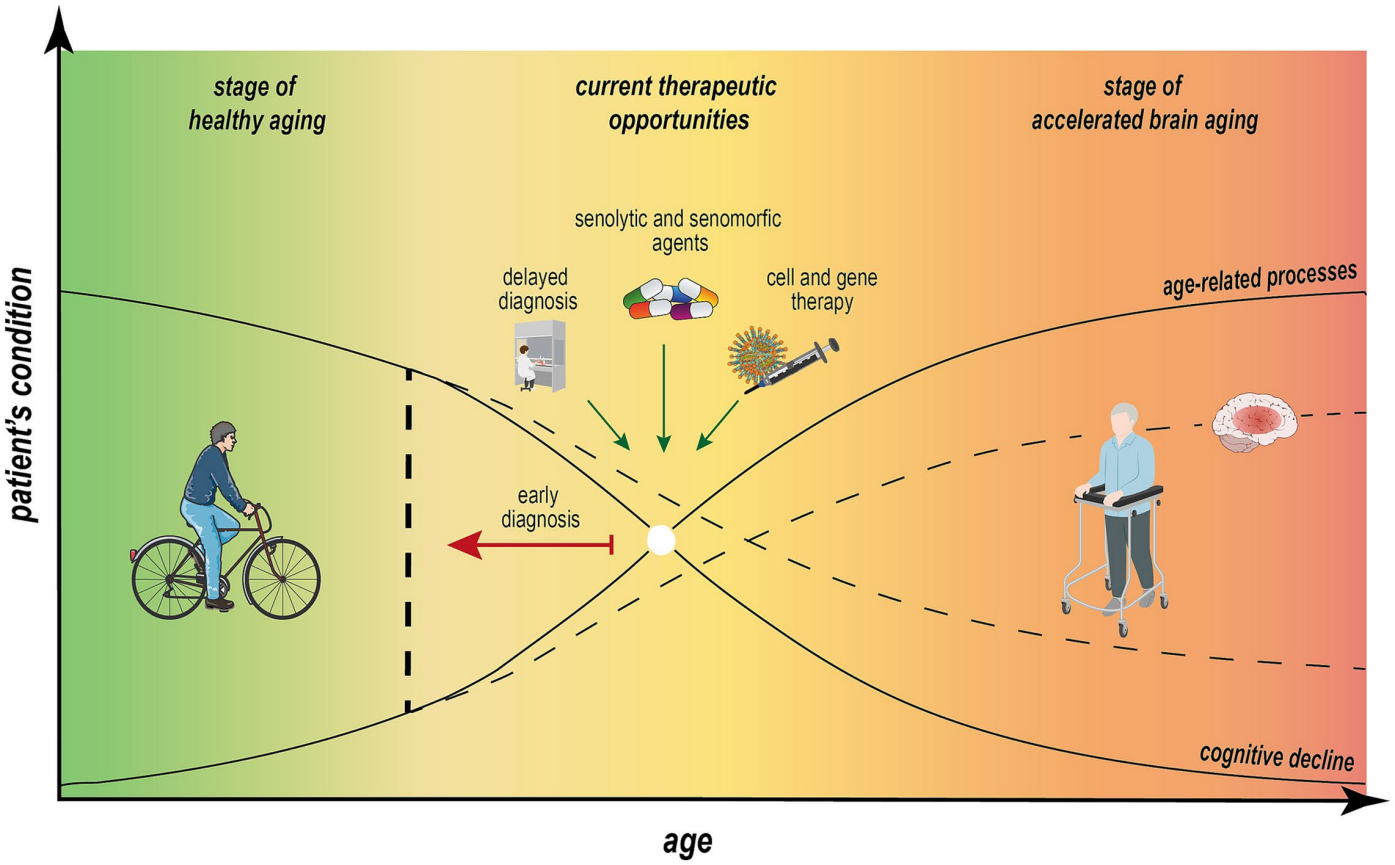
Nonlinear dependence of aging processes and cognitive impairment across individual aging trajectory. Estimating of predicted age difference (PAD) between measurable “brain age” and chronological age is essential to define individual aging trajectory. The progression from healthy aging to accelerated brain aging, ultimately leading to dementia or neurodegenerative diseases, is influenced by physical, genetic, and social factors. This trajectory is marked by a significant decline in cognitive function in parallel with impairments in metabolic processes, neuroinflammation, and longevity. Early diagnosis through molecular patterns, behavioral tests, and advanced imaging systems, combined with therapeutic interventions, can positively impact the mid-stage of this trajectory, which is potentially reversible. Implementing these strategies may mitigate age-related processes, reduce the rate of cognitive decline, and promote the sustainability of cognitive and overall health in aging individuals.

The development of effective therapeutic strategies for neuroinflammation and related age-associated diseases is an area of intense research. Various laboratory models, including those induced by A*β*(1–42) oligomers, D-galactose, lipopolysaccharides, or specific cell lines, have been utilized to explore potential treatments (Table 2). High priority is given to understanding the correlation between biomarker levels and therapeutic efficacy (Figure 3) (Liu Y. et al., 2020; Torres et al., 2022; Zeng et al., 2021). One promising approach is senolytic therapy, which aims to eliminate senescent cells. Torres et al. investigated the effects of senolytics, such as navitoclax and the dasatinib–quercetin combination, in an age-related disease model of amyotrophic lateral sclerosis. Their study demonstrated a reduction in senescence biomarkers, including p16, p21, SASP, and SA-β-gal (Torres et al., 2022). Similar reductions and improvements in cognitive function were observed in APP/PS1 transgenic mice, a model for Alzheimer’s disease, as well as in INK-ATTAC transgenic mice (Ogrodnik et al., 2021; Zhang et al., 2019).

In contrast, senomorphic agents target and regulate the pathways involved in cellular senescence rather than removing senescent cells (Di Micco et al., 2021). These agents work by modulating the expression of specific genes or proteins to mitigate the detrimental effects of senescent cells (Di Micco et al., 2021). Rapamycin is an example of a senomorphic agent studied for its potential anti-aging properties, particularly through mTOR inhibition. Research has shown that rapamycin exerts an anti-inflammatory effect by targeting LPS-induced neuroinflammation in N9 microglia and in the hippocampus and cortex of mice. This treatment results in a decrease in pro-inflammatory cytokines (TNF-*α*, IL-1β, and IL-6) and an increase in anti-inflammatory IL-10 secretion (Ye et al., 2020).

Despite the significant role of inflammation during the accelerated brain aging, the nonsteroidal anti-inflammatory drugs (such as NSAIDs) have not demonstrated definitive results. Challenges such as unclear dosing, poor blood–brain barrier (BBB) permeability and and the potential for autoimmune side effects contribute to their limited effectiveness (Kalra et al., 2022). Although inflammation response was reduced in the *nfkb1−/−* mouse model, cognitive dysfunction persisted in patients with AD and MCI (Fielder et al., 2020; Rivers-Auty et al., 2020). For these reasons, as well as the potential for undesirable side effects following prolonged treatment with NSAIDs, the searching of potential possibilities for targeting to the aging process is necessary.

At the present time, there is a considerable increase in the number of studies being conducted on the balance between microglial cells of type M1/M2. The activation of the M1-like phenotype is associated with the secretion of pro-inflammatory cytokines and chemokines, including TNF-α, IL-6, and IL-18. In contrast, the M2 type of microglia has been shown to have anti-inflammatory effects (Wang et al., 2023). It was demonstrated that both biological aging and a stressful aspect (e.g., peripheral surgery or traumatic brain injury) alter the balance in favor of the pathological M1 phenotype induction (Zhang Z. et al., 2020). Wu et al. (2021) determined that initiation of myeloid-epithelial-reproductive tyrosine kinase (Mer)-signaling increases the emission of markers (CD206, Arg-1, IL-10) with an M2-related microglial cells in the TBI mouse model. Inhibition of Mer-signaling with siRNA leads to the deterioration of the functional outcomes following TBI, with the rise in the number of neuronal degenerations in the lesion and a reduction in the expression of molecules of downstream cellular pathways (SOCS-1/3). In contrast, the infusion of the Mer-activator protein S represses the markers CD16, CD32, and iNOS of M1-like phenotype and switch on the anti-inflammatory effect of the M2-type (Wu et al., 2021). Similar results in the field of microglia phenotype shifting to M2 polarization were observed in the LPS-induced mouse model of neuroinflammation (Liu J. et al., 2020). The decreased nuclear translocation of NF-κB in a dose-dependent way, induced by ginsenoside Rg1, has shown the tissue repair, secretion of anti-inflammatory cytokines and a neuroprotective role, making the NF-κB pathway perspective for the future researches.

In the context of the senescent cells’ growth, neurogenesis decline and the long-term action of the M1 pro-inflammatory microglia during accelerated brain aging, it is necessary to reprogram cells into a healthy state in order to reduce the pathogenic effect. Most of studies focused on the reversing age-related changes and promoting tissue regeneration. It was verified that the influence of the Yamanaka transcription factors (OSKM) on the SVZ neurogenic niches and the entire body in groups of old mice (at 18–20 months and 24–26 months) results in an increase in the rate of neuroblasts and their precursors (Xu et al., 2024). Furthermore, the knockout of Ptbp1 mRNA by CRISPR/CasRx provide to get the pull of dopaminergic neurons from the glial cells in the 6-OHDA-induced mouse model of Parkinson’s disease *in vivo*. Additionally, it was observed that this type of effective reprogramming of MG to functional neurons in the striatum alleviates motor dysfunction in a model of PD (Zhou et al., 2020). Another crucial area of investigation is the reprogramming of senescent cells in neuronal tissue. The reversal of cellular senescence may be a means of restoring proper function and reducing neuroinflammation associated with aging.

Besides microglia, there is another population of glial cells that demonstrates high morphological and behavioral variability (Khakh and Sofroniew, 2015; Cunningham et al., 2019; Sofroniew, 2020). It had long been thought that the primary functions of astrocytes are the maintenance of homeostasis, neuronal bioenergetics, and cell communication. The present studies had recorded the reactive state of astrocytes that change the transcriptomic profile, morphology and function under various pathological triggers (Sofroniew, 2020). Accordingly, a generalized subset of astroglia phenotypes has been taken to be divided into neurotoxic state that activated by pro-inflammatory factors of microglial cells, and neuroprotective phenotype (Escartin et al., 2021). However, these bipolar states are not characterized the overall phenotypes of astrocytes in several disorders. In connection with accelerated brain aging, Liddelow et al. (2017) found the activation of neurotoxic status of astrocytes marked with complement component C3 by the concurrent secretion of Il-1α, TNFα and C1q whose levels increase due to microglial activation *in vitro* and *in vivo*. This allows astrocytes to express neurotoxin that contributes to the destruction of neurons and oligodendrocytes and decreased stability of synaptic contacts (Liddelow et al., 2017). In addition, the transcriptome analysis by RNA sequencing (RNAseq) was performed for astrocytes from three brain regions (the hippocampus, striatum and cortex) at five different ages over the lifespan of mice (Clarke et al., 2018). It was found the phenotype heterogeneity of cells according to the region-specific changes during the normal aging. Thus, the cells derived from old mice (2 years old) in the hippocampus and striatum show a more reactive profile toward the neurotoxic state which leads to cognitive decline (Clarke et al., 2018). This condition is induced by the pro-inflammatory mediators from microglia and increased levels of peripheral immune cells in the brain which also secrete the cytokines. So, the combined effect on the glial cells (in particular on microglial cells and astrocytes) to shift the phenotype towards the neurotrophic and neuroprotective status could be one of the promising ideas for the study of aging in norm and pathology.

Moreover, not only the therapeutic approaches can impact on the neuroinflammation rate during the aging. Healthy nutrition, especially caloric restriction (CR), promotes the normal neural density in the hippocampal hilus, also as decreases the inflammatory response in comparison to the rats’ group with unlimited access to food (*Ad Libitum* (AL) group) (Portero-Tresserra et al., 2023). Although, there are no differences in neurogenesis in CR and AL groups (24 months). The nutritional approach under discussion here contributes to neuronal activity and synaptic plasticity and normal mitochondrial functions, confirming the reduction of cognitive deficits (Lin et al., 2014). It supports the hypothesis that genetic factors are crucial for their potential clinical applications, such as early detection of brain aging and relevant interventions, however, the healthy diet and physical activity are equally important for normal aging.

## 8 Concluding remarks and future perspectives

Defining individual aging trajectories has become increasingly critical as societies face the challenges of an aging population. The conventional methods of assessing aging, which include neuroimaging, cognitive testing, and genetic analysis, have provided valuable insights. However, these methods alone may not fully capture the complexities of individual aging. Integrating advanced technologies, such as AI and digital twins (DT), offers promising avenues for more precise and personalized aging assessments.

DT are also becoming essential tools in healthcare, marking a significant advancement at the intersection of technology and translational medicine (Woolf, 2008). Digital twins are virtual models that simulate and monitor the health and aging processes of individuals in real time. The concept of DT, particularly in the context of aging, represents a significant advancement in healthcare technology. DT can represent anatomical structures or model dynamic processes, especially in disciplines such as neuroscience, where they simulate brain function and predict clinical outcomes. As virtual representations of physical systems, DT enable modeling, comprehensive analysis, and prediction. They are also finding increasing interest and applications in healthcare, with a particular focus on digital twins of the brain (Fekonja et al., 2024). This approach can provide a more nuanced understanding of aging trajectories by incorporating data from neuroimaging, cognitive tests, and genetic profiles into a cohesive model. For instance, the e-VITA project exemplifies the potential of digital twins to create a virtual coaching system for the elderly, which could serve as a model for understanding and managing aging (Lehmann et al., 2024). With interdisciplinary efforts, estimating individual aging trajectory has the potential to transform healthcare system and contribute to a healthier aging.

The technical background, suggesting that the brain’s nonlinear behavior toward enhancement and recovery can be modeled and predicted from fMRI data, exploits the brain’s dual capacity for adaptive and disruptive plasticity. DT can model the brain’s response to injury and pathology, helping to bridge the gap between theoretical research and clinical practice (Fekonja et al., 2024). Thus, in medical and neuroscience disciplines, DT embody the interplay between virtual models and biological reality. Their adaptability not only enhances our understanding of complex physiological phenomena, but also allows us to reflect on the theoretical concepts underlying phenomena such as aspects of intelligence, consciousness, plasticity and adaptability.

Integrating all these layers of information into a unified algorithm holds tremendous promise. Such an advancement could enable early detection of individuals at risk for accelerated brain aging, allowing for targeted interventions to delay cognitive decline. In summary, the convergence of comprehensive genomic profiling, deep phenotyping, and advanced computational models is poised to revolutionize our approach to brain aging, making personalized medicine a tangible reality in the near future.

Furthermore, deep learning techniques have demonstrated the ability to map brain aging trajectories at an individual level, incorporating multimodal data such as neuroimaging, genomic profiling, and clinical parameters. This approach enables early identification of individuals at risk for cognitive impairment, facilitating preclinical intervention strategies (Jung et al., 2024). The integration of AI-driven models with biological age predictors, including transcriptomic and proteomic markers, enhances the precision of aging assessments and provides insights into personalized therapeutic approaches. By leveraging advanced computational techniques, future research can refine predictive algorithms to distinguish between normal and pathological aging, ultimately contributing to early detection and targeted interventions for neurodegenerative diseases.

## Glossary

AD: Alzheimer disease
AS: atherosclerosis
BA: biological age
BBB: blood–brain barrier
CR: caloric restriction
DT: digital twins
GWAS: genome-wide association studies
KDM: Klemera and Doubal’s method
LPS: lipopolysaccharide
MLR: multiple linear regression
MRI: magnetic resonance imaging
MS: multiple sclerosis
NSC: neural stem cell
PAD: predicted age difference
PCA: principal component analysis
PD: Parkinson disease
ROS: reactive oxygen species
SASP: senescence-associated secretory phenotype
SC: senescent cell
SGZ: subgranular zone
SVZ: subventricular zone
